# Enabling Eating Detection in a Free-living Environment: Integrative Engineering and Machine Learning Study

**DOI:** 10.2196/27934

**Published:** 2022-03-01

**Authors:** Bo Zhang, Kaiwen Deng, Jie Shen, Lingrui Cai, Bohdana Ratitch, Haoda Fu, Yuanfang Guan

**Affiliations:** 1 Eli Lilly and Company Indianapolis, IN United States; 2 University of Michigan Ann Arbor, MI United States; 3 Ann Arbor Algorithms Ann Arbor, MI United States

**Keywords:** deep learning, eating, digital watch

## Abstract

**Background:**

Monitoring eating is central to the care of many conditions such as diabetes, eating disorders, heart diseases, and dementia. However, automatic tracking of eating in a free-living environment remains a challenge because of the lack of a mature system and large-scale, reliable training set.

**Objective:**

This study aims to fill in this gap by an integrative engineering and machine learning effort and conducting a large-scale study in terms of monitoring hours on wearable-based eating detection.

**Methods:**

This prospective, longitudinal, passively collected study, covering 3828 hours of records, was made possible by programming a digital system that streams diary, accelerometer, and gyroscope data from Apple Watches to iPhones and then transfers the data to the cloud.

**Results:**

On the basis of this data collection, we developed deep learning models leveraging spatial and time augmentation and inferring eating at an area under the curve (AUC) of 0.825 within 5 minutes in the general population. In addition, the longitudinal follow-up of the study design encouraged us to develop personalized models that detect eating behavior at an AUC of 0.872. When aggregated to individual meals, the AUC is 0.951. We then prospectively collected an independent validation cohort in a different season of the year and validated the robustness of the models (0.941 for meal-level aggregation).

**Conclusions:**

The accuracy of this model and the data streaming platform promises immediate deployment for monitoring eating in applications such as diabetic integrative care.

## Introduction

### Background

The technological progress of wearable devices, such as smartwatches and wristbands, has made them an integral part of our lives [1]. Wearable devices provide rich, high-frequency, and longitudinal information for symptoms or activities relevant to improving patient diagnosis, care, and treatment. Being able to identify specific relevant activities, such as food intake, in a way that places a minimal burden on that person has the potential of increasing efficiency of monitoring and patient satisfaction. For example, current diabetes management using basal and bolus insulin regimens requires a high level of patient engagement. One-third of patients with type 1 or type 2 diabetes report insulin omission or nonadherence at least once in the past month, and one of the cited reasons is being too busy [2]. In this scenario, passively collected digital sensor data from consumer wearable devices could be an ideal approach for supplementing the sensor and patient-provided data collected by specialized connected care diabetes devices. Apart from diabetes, a variety of diseases have been linked to poor eating habits, including heart diseases, obesity, high blood pressure, and other leading causes of death [3,4]. The ability to monitor eating behavior on a continuous basis is central to improving the care and treatment of these conditions.

### Related Works

The current literature includes studies of automatic food intake detection using a variety of sensors (Table 1), such as audio, motion, and specialized sensors for chewing and swallowing detection, mounted on different parts of the body such as wrists, head, ears, and neck [5-11]. Although published results are encouraging and indicate the feasibility of automatic food intake detection, advancement in data collection and analytics is still in need. First, most of the existing studies have been conducted in the laboratory [12-15], whereas data on eating in the free-living environment is more difficult to obtain and infer. Second, if a study is conducted in a free-living condition, it is challenging to obtain accurate ground truth. Typically, such ground truth is obtained through food diaries or questionnaires, and the failure to memorize eating times impedes establishing accurate models [16,17]. Third, because of the cost of wearable watches, participant recruitment, and data extraction, pioneering studies so far are very limited in size, typically covering dozens to hundreds of hours of records in total (Table 1) [18]. For example, Farooq and Sazonov [19] took a total of 23 hours of records >10 individuals in a free-living environment to study the effectiveness of accelerometers in detecting eating. A study that is comparable in size to this one is the Sharma et al [9] study, which contained 1413 hours of records. Finally, this study distinguishes itself from the above studies by its longitudinal follow-up of weeks. This allowed us to update the models for each device user as the data collection proceeded.

**Table 1:** Representative literature with relatively large size of data on eating detection.

Study			Definitions of eating		Device position		Number of participants		Total hours		F1 score (%)		Weighted accuracy (%)
Dong et al [7]			Daily meals and snacks		Wrist		43		449		N/A^a^		81
Thomaz et al [8]			Laboratory: participants were asked to use a fork, knife, hand, and spoon to eat lasagna, popcorn, sandwich, breakfast cereal, rice, and bean Free-living: normal daily meal activities		Wrist		8		784.25		76.1 and 71.3		N/A
Sharma et al [9]			A complete meal or snack		Wrist		104		1413		N/A		75
Zhang and Amft [20]			Participants had no constraints on diet selection and daily activities. They were asked to manually log every eating event in a diet journal of a 1-minute resolution.		Eyeglasses		10		122.3		95.2^b^		N/A
Bi et al [11]			Laboratory: 6 types of food with 3 crunchy types and 3 soft types Free-living: daily meal activities		Ear		14		32.2		77.5		92.8
Zhang et al [10]			An aggregate of chewing sequences that occur within a short duration of time; these chewing sequences are separated from other chewing sequences by a large time gap		Neck		20		370.1		81.6		N/A
Farooq et al [19]			N/A		Eyeglasses		10		23		87.9		N/A
**This work**													
	5-minute chunks	N/A		Wrist		34		3828.25		93.8		78	
	Whole meals in the discovery cohort	N/A		Wrist		34		3828.25		87.7		88	
	Whole meals in the validation cohort	N/A		Wrist		34		3828.25		87		87	

^a^N/A: not available.

^b^Best.

### Objective

Our objective is to develop a prospective, noninterventional, observational study that addresses the above challenges in detecting events of food intake based on passively collected motion sensor data from wearable devices in free-living conditions. We also aim to test the performance of the deep learning algorithms in detecting eating using this data. To this end, we developed a specialized app that allows the recording of eating diaries by simply tapping on the smartwatch and automatic streaming of the accelerometer and gyroscope data into the cloud computing platform. A total record of 3828.25 hours (1658.98 in the discovery cohort and 2169.27 in the validation cohort), encompassing 6 types of eating utensils (forks, knives, spoons, glass, chopsticks, and hands), provided us with deep data for developing models that infer eating behavior in the general population. We develop models that have an area under the curve (AUC) of 0.951 for detecting an entire meal event. We also show the potential to fine-tune more accurate personalized models. A prospective, independent cohort further validated the model. The accuracy of this model supports its immediate readiness to be deployed in clinical trials such as connected diabetes care devices and other therapeutic areas.

## Methods

### Recruitment and Ethics Approvals

The inclusion criteria of participants in the study were as follows: (1) aged ≥18 years; (2) living in the United States; (3) an Eli Lilly employee working in a Lilly office in Indianapolis, United States; (4) willing to wear an Apple Watch, which is provided for this study and which will be used to collect data from the device motion sensors and logs of events of food consumption; (5) owning a Lilly iPhone and willing to pair it with the Apple Watch provided in this study and to use an app developed for this study to facilitate transfers of motion sensor data; (6) having an internet connection with access to a secure password-protected Wi-Fi at home for the duration of the study; and (7) willing to not use another wrist-worn personal device (eg, Apple Watch) for the duration of this study. The exclusion criteria were as follows: (1) experiencing from hand tremors or involuntary arm movements, (2) currently being a smoker, (3) participation in any other study involving wearable devices that may interfere with the conduct of this study at any point during participation in this study, and (4) being involved in the planning or conduct of this study or being a member of the Machine Learning and Artificial Intelligence team of the Advanced Analytics and Data Sciences group at Eli Lilly. The study has been approved by the Eli Lilly Review Board (study number: 2019-8193) and reviewed by the Western Institutional Review Board (WIRB protocol number 20190878), and all participants have provided written consent to this study. The informed consent form is provided in Multimedia Appendix 1.

### Instruments

The purpose of this study is to develop a data streaming system and algorithms that could automatically collect and detect eating events based on passive monitoring of motion sensor data from wearable devices in free-living conditions. The terms used throughout this paper are outlined in Textbox 1.

**Terminology and notations used in the paper.**

**Terms and explanations**

- Window
  A segment from the data used as the model input (typically 5 minutes in this study)
- Moving step
  The size of the stride between 2 consecutive windows
- Session
  A session is a consecutive recording from the watch; a single day can have multiple sessions.
- Region
  A segment of data within a specific time range
- Aggregation
  The methods that we use to determine the inference of a region based on its related windows
- n
  A cutoff helping to determine if a meal region is inferred correctly or if a region is false positive
- False positive regions
  A region containing at least n false positive data windows
- (Hourly) false positive detection rate
  (Number of the false regions–number of the positive regions)/total sample hours

**Figure 1:**
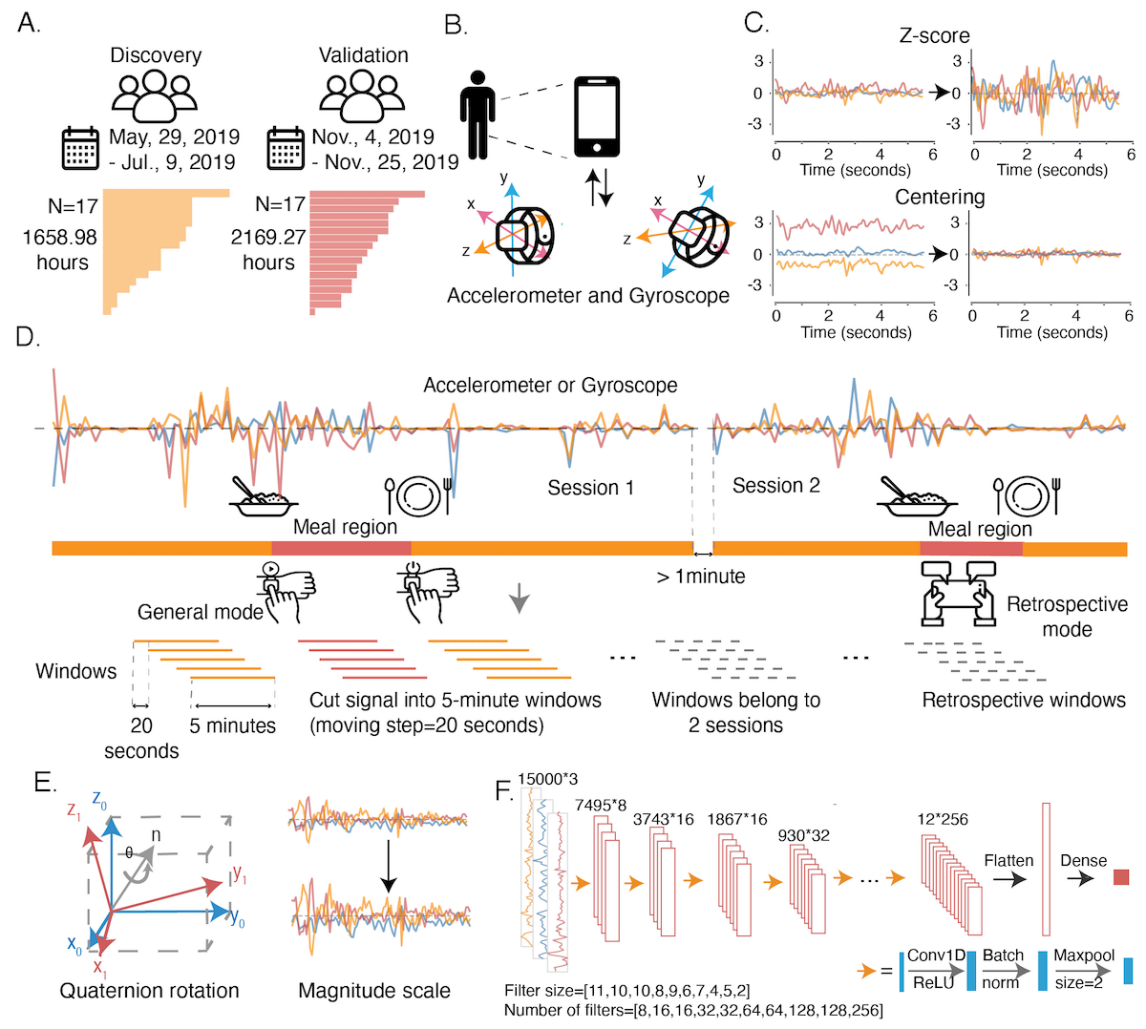
Overview of data collection and streaming for meal activity analysis. (A) Data comes from two cohorts: 17 participants in the discovery cohort with 1658.98 hours of data and 17 participants in the validation cohorts with 2169.27 hours of data. (B) Signals were collected by 2 sets of sensors, accelerometer and gyroscope, in Apple Watch and paired with iPhone. (C) Z score and centering normalization were conducted for each of the x, y, and z axes for each window. (D) The gyroscope and accelerometer provide continuous signals on the x, y, and z axes over time. There are 2 modes to record meal time. The general model recording starts and stops by tapping the button on the Apple Watch. Retrospective mode allows the participant to type in the rough meal time with the iPhone after having a meal. For each record, we took 5-minute windows with a moving step of 20 seconds. The windows with >2.5 minutes of mealtime will be labeled as eating activity. Windows belonging to 2 sessions are removed. (E) Two augmentation methods, quaternion rotation and scaling the signal magnitude, apply to each data window. (F) Deep learning network structure.

Our goal is to detect eating activity based on data from motion sensors embedded in wearable devices to minimize the risk of privacy invasion. Eating activity in humans involves potentially distinguishable movements—hand-to-mouth gestures. A total of two motion sensors—accelerometer and gyroscope—were used for the position and orientation sensing in digital watches (Figure 1B). We used the Apple Watch Series 4, which is equipped with both sensors. We programmed the watch to extract sensor data using a standard application programming interface (API), which can be seamlessly paired with an iPhone to facilitate data flow and retrospective labeling. This study has been approved by the Eli Lilly Western Institutional Review Board, and participants’ information was deidentified before analytic research. Eating dairy is in general recorded by 2 simple tappings of *begin* and *end* on the Apple Watches. Compared with previous eating diaries, this method facilitates accurate recordings of the eating region.

Participants were asked to log all events of food intake regardless of the type of food or beverage consumed or the manner in which it is consumed (eg, with or without utensils, sitting down, standing, or walking), except while driving. Specifically, they were asked to log each region of food intake if she or he estimated that it would involve >3 bites or sips (movements to bring the food to one’s mouth) and would last for >2 minutes. Activities such as taking oral medications, using chewing gum, or taking a few sips of water did not need to be logged. Although many people wear a watch on a nondominant arm, in this study, we asked the participants to wear the study Apple Watch on the arm that they consider dominant for eating purposes. This choice is motivated by the available literature indicating that the food intake detection algorithms using motion sensor data from the dominant arm provide better performance than those using data from the nondominant arm, whereas using sensor data from both arms does not improve performance significantly [5].

We recruited 2 independent cohorts. The first cohort included 17 individuals, deidentified before data analysis—02CE, 064F, 08A5, 0D51, 0FA7, 11FD, 1453, 16A9, 1B55, 2257, 2BAF, 305B, 32B1, 375 D, 3e5f, 4561, and 47B7—and spanned between May 29, 2019, and July 7, 2019. The second cohort included 17 individuals—766F, 7D71, 7FC7, 8473, 94CD, 9979, A07B, AE7F, B0D5, BED9, C385, CA87, D189, D3DF, DF8D, E1E3, and E68F—and spanned between November 4, 2019, and November 25, 2019. For each participant, we longitudinally collected a maximum of 20 (discovery cohort) and 22 days (validation cohort) of their daytime activities, with a median of 9 and 11 days, respectively. This provided a total of 1658.98 hours of data in the discovery cohort and 2169.27 hours of data in the validation cohort (Figure 1A). The discovery cohort included 162 days of samples in total, where each individual was allowed to take different numbers of days of experiments varying from 1 (eg, participant 1453) to 20 (eg, participant 2257; Multimedia Appendix 2, Figure S1). The validation cohort included 193 days of samples in total, with the experiment days varying from 1 (eg*,* participant A07B) to 22 (eg*,* participant CA87; Multimedia Appendix 2, Figure S1).

Data were recorded at a frequency of 50 Hz and were segmented into individual files by the combination of collection date and participant ID. It is common to see multiple sessions in a single file (Figure 1D and Textbox 1), corresponding to consecutive recording periods in a single day. Approximately 25.3% (41/162) of the samples contain >1 session in the discovery cohort, and approximately 68.9% (133/193) of the samples in the validation cohort have at least two sessions (Multimedia Appendix 2, Figure S1C). The data are presented in a timewise fashion of 20 features, including acceleration and rotation rate (*accl_x_* for acceleration at the x-axis, *accl_y_* for acceleration at the y-axis, *accl_z_* for acceleration at the z-axis, *gyro_x_* for gyroscope at the x-axis, *gyro_y_* for gyroscope at the y-axis, and *gyro_z_* for gyroscope at the z-axis), utensils (binary labels of utensils, fork, knife, spoon, glass, chopstick, and hand), ground truth labels (*tag* for all eating tags, *tagTimely* for eating tags that are done when eating happens, and *tagRetro* for retrospectively recorded taggings), session (*sesid*), timestamp (*ts*), and the local time (*tod*). We determined whether a positive tag should be considered in the training by tagTimely, a binary feature indicating whether the tag is labeled during mealtime.

The data collection platform will also enable the participants to retrospectively log approximate times of meals if they forget to log them in a timely manner. The choice of collecting ground truth classification labels through participants’ logs is also motivated by the fact that a potential future activity detection system deployed in real life may collect some amount of personalized training data to fine-tune the inference model to individual characteristics.

### Data Cleaning

A couple of noncompliances appeared to have come from the misunderstandings of the guidance. For example, participant 4561 presented very short sampling regions on June 6, 2019 (Multimedia Appendix 2, Figure S2A). It appears that she/he recorded only the mealtime. Another noncompliance was observed in the close-to-zero signals in accelerometer and gyroscope data for a long region of time; for example, 47B7_2019-06-07 (Multimedia Appendix 2, Figure S2B). This identification number follows the format of participant ID_date. It is likely that participants took off their watch during these time regions. We further removed individuals or days without eating records (eg, 2257_2019-06-27). Communications with participants indicated that they were incorrectly annotated.

**Figure 2:**
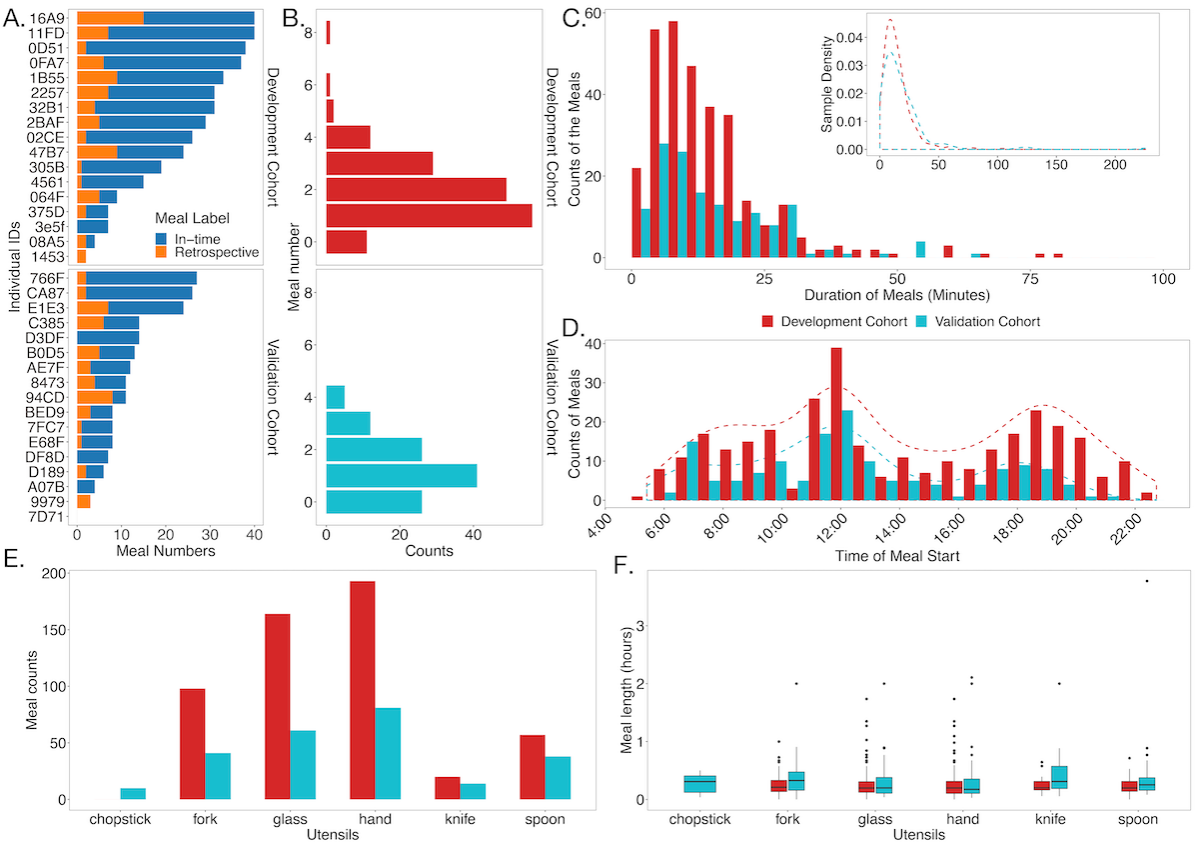
Summary of the meal regions. (A) Describes the meal number of each individual in the discovery cohorts and the validation cohorts. The bars are sorted by the total number of the meals and comprise two types of model labeling: label during eating (in-time, the blue bars) and label by retrospect (retrospective, the orange bars). (B) Summarizes the meal numbers per day excluding the retrospectives. (C) Shows the distributions of the duration (in minutes) of the meals for the discovery and the validation cohorts, excluding the retrospective meals. (D) Plots the distributions of the starting times of the meals in these 2 cohorts, and the dashed lines correspond to smoothed curves describing the counts. (E) Distribution of the numbers of the meals with different utensils in the discovery and the validation cohorts. (F) Statistics of the meal lengths (in hours) for different utensils in the discovery and the validation cohorts.

For the discovery cohort, participants 064F, 1453, and 08A5 were excluded from this study because of an overwhelming number of retrospectively annotated meals (>50%; Figure 2A), which indicates potential poor data quality of these days. Records from these participants are also removed as the sampling times are <3 hours in a day, as they are possibly not compliant with the instructions that require wearing the watch during day time: 4561 (May 30, 2019, and June 6, 2019), 305B (June 11, 2019), 47B7 (June 7, 2019, and June 1, 2019), 2257 (June 27, 2019), 375 D (June 7, 2019), 32B1 (June 18, 2019), and 0D51 (June 11, 2019). Of the 17 individuals, 14 (82%) individuals remained to consider in the model development for the global model. The personalized models were fine-tuned and evaluated on the 86% (12/14) of individuals with ≥7 samples: 02CE, 0D51, 0FA7, 11FD, 16A9, 1B55, 2257, 32B1, 2BAF, 305B, 4561, and 47B7.

For the validation cohort, based on the same exclusion rules, we also kept 82% (14/17) of individuals and removed participants 9979 (3/3); 94CD (8/11), who had an overwhelming number (>50%) of retrospectively recorded meals; and 7D71 (0/0), who failed to label the meal times in all dates. Then, we removed 15 person days with a total duration of <3 hours and another 55 person days, which did not contain any meal times.

### Data Preprocessing

All the data were cut into 5-minute (300 seconds) windows with a moving step of 20 seconds from the start of each date of data based on the record length. The label for each 5-minute data window was determined by the proportion of mealtimes: if the window has >2.5 minutes (150 seconds) labeled as mealtime, then the label of the window is 1 (positive examples); otherwise, it is 0 (negative examples). There were 3 additional conditions that generated −1 labels, which were excluded in both training and evaluation (Figure 1D). The first one is when the current window belonged to 2 different sessions; the second is that the window included the records whose tagRetro (retrospectively recorded eating) was not 0 or missing, which means these tags were recalled by the users after their meals. Third, extremely short periods of eating <3 minutes were excluded as they could have disrupted the fairness of evaluation. A total of 282,942 windows were generated according to this preparation method from the discovery cohort, and 13,498 of them were positive. As the data were highly unbalanced, we applied an oversampling: we randomly selected N records from the positive examples with replacement, where the N is the number difference between the negative examples and the positive examples.

### Model Training and Evaluation

The general training and evaluation strategy was cross-validation, a commonly used scheme that ensures sufficient test examples. In each test, we randomly selected 21% (3/14) of individuals for the test set, 18% (2/11) of individuals for the validation set, and the rest 64% (9/14) of individuals as the training set. Models were trained and tuned on the training and the validation sets, and we evaluated the performances on the test set. We also trained 5 models for each test, which came from 5 random splits on the training set and the validation set while maintaining the 3 final test individuals unchanged. The final inference scores for the evaluations were the averaged ensemble of the 5 models.

We experimented separately based on accelerometer and gyroscope data, and then we assembled the inference scores using the 2 types of data by taking the average. Experiments are organized in the following order: input data, normalization methods, and augmentations. In each step, we selected the best-performing model for the next experiment. For the fine-tuned personalized model, we first trained a model using all the data in the discovery set, excluding the individual that was the target of fine-tuning. Then, we fine-tuned the model for 2 additional epochs on 60% of the days of the target individual, using another 20% of the days as validation and the last 20% of the days as the test set. Across all experiments, the evaluation was conducted on the testing set with the original class imbalance.

### Model Architecture

The backbone of the models is a deep convolutional neural network, comprising 10 building blocks and a fully connected layer for the output listed in Multimedia Appendix 2, Table S13 and Figure 1F. Each block contains a convolutional layer, a batch normalization layer, and a maxpooling layer. The number of filters grows progressively from 8 to 256 (8,16,16,32,32,64,64,128,128, and 256). The sizes of the filters follow (11,10,10,8,9,6,7,4,5, and 2). The network receives both the 3-channel inputs from the accelerometer or the gyroscope and the 6-channel inputs when using them together. The weights of the network are trained by an Adam optimizer [21], the most popular parameter optimizer, with a learning rate of 0.00003 and a binary cross-entropy loss function as the training target is binary. To combat overfitting, we applied a callback function to retrieve the weights from the epoch of the best performance on the validation set. These selected weights were then applied to the test set to evaluate the model performance. We trained a total of 5 epochs. The kernel was initialized with Glorot uniform. The abovementioned parameters were selected empirically and then searched around the empirical values.

### Normalization and Data Augmentation

We tested two normalization methods: centering and *z* score normalization (Figure 1C). The outputs of centering were the subtraction between the original values and the averages, and the *z* score normalization required a calculation based on the following formula, where the µ is the average, and the σ is the SD.



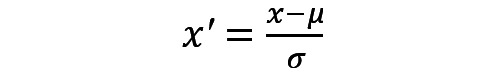



To combat overfitting, we applied 2 augmentation methods (Figure 1E). The first was scaling the signal magnitude by multiplying a randomly selected number from a uniform distribution in (0.8, 1.2). The second was rotating the signals by multiplying a quaternion rotation matrix, which mimics the situations where the same record is taken in different reference frames. First, we randomly generated a set of coordinates (x, y, and z) and defined a reference frame by calculating the basis vectors.



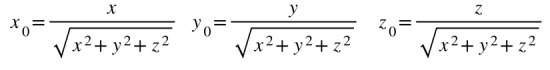



Then, we randomly seeded a rotation angle from (0, 2π), and calculated as follows:



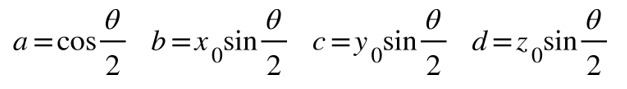



The rotation matrix, which multiplies to the original acceleration or gyroscope signals, were then defined as follows:



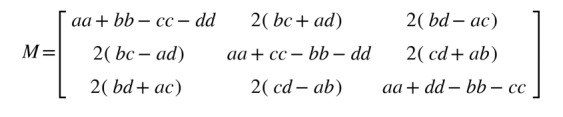



### Evaluation Metrics

The model performances were evaluated on the ensembled inference scores and by a series of metrics, including the area under the receiver operating characteristics curve and the area under the precision–recall curve (AUPRC). Using the information of true positive *TP*, true negative *TN*, false positive *FP*, and false negative *FN*, we evaluated the weighted *F*_1_ score and the relating precision and recall scores [22], where *i* is the index of the class, and the w_i_=number of class *i* samples/total sample numbers is the proportion of the class *i*.



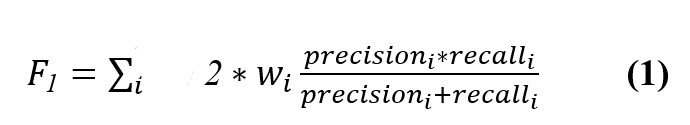



Precisions came from TP/(TP+FP), and recalls came from TP/(TP+FN). We also calculated weighted accuracy following the method in the studies by Dong et al [7] and Sharma et al [9]:



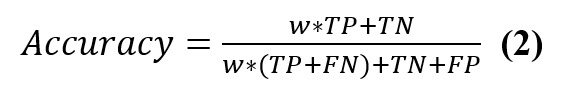



where *w* is the ratio of the number of negatives over the number of positives.

All the metrics were calculated by the corresponding functions in scikit-learn.

### Comparison With DeepConvLSTM

We applied DeepConvLSTM [22] based on the official Pytorch implementation. We used a filter size of 32, and the number of hidden units in the long short-term memory was 64. Details of the structure and the parameters are listed in Multimedia Appendix 2, Table S14. We used the Adam optimizer and binary cross-entropy loss function.

### Statistical Significance Analysis

For model comparison, in each test of inference, we first calculated the ratio (denoted as *R*) of the positive (label=1) to the negative examples (label=0) and then randomly selected 1500 positive 5-minute windows and *1500R* negative windows. We repeated 100 times to estimate the P values.

### Code Availability

The code is attached with the submission (Multimedia Appendix 3) and can be runnable with Python 3.6.12 and Keras 2.2.4.

### Data Availability

On the basis of consent forms, Eli Lilly can share data with regulatory authorities (Food and Drug Administration) in the United States, the ethical review board overseeing this study, and the researchers at other institutions who wish to analyze the data in this study.

## Results

### Deep Learning Accurately Classifies Eating Activity in 5-Minute Windows on Previously Unseen Individuals

We first analyzed the data from the discovery cohort. Raw data were collected from the accelerometer and gyroscope from the Apple Watch at a frequency of 50 Hz and streamed to Amazon Web Service [23]. Each time point was labeled with a meal tag (1 denoted the meal region and 0 denoted non–meal time). Participants were asked to specify the start and the end of the mealtime and whether this region was recorded at the time of the meal or retrospectively. Most (126/162, 77.8%) of the daily records lasted approximately 8 to 15 hours, representing the daily activity time when the participants wore the watches (Multimedia Appendix 2, Figure S1B). The participants are likely to begin their records from 7 AM to 9 AM (Multimedia Appendix 2, Figure S1D) and end at 7 PM to 9 PM (Multimedia Appendix 2, Figure S1E), which is consistent with the expected daily activity time. A participant could have 1 to 7 eating events within a day, with the vast majority having between 1 and 4 eating events per day. Approximately 75% of the meals would last for <20 minutes. The start and peak times of the meal events were shown at the expected breakfast, lunch, and dinner times (Figure 2).

**Figure 3:**
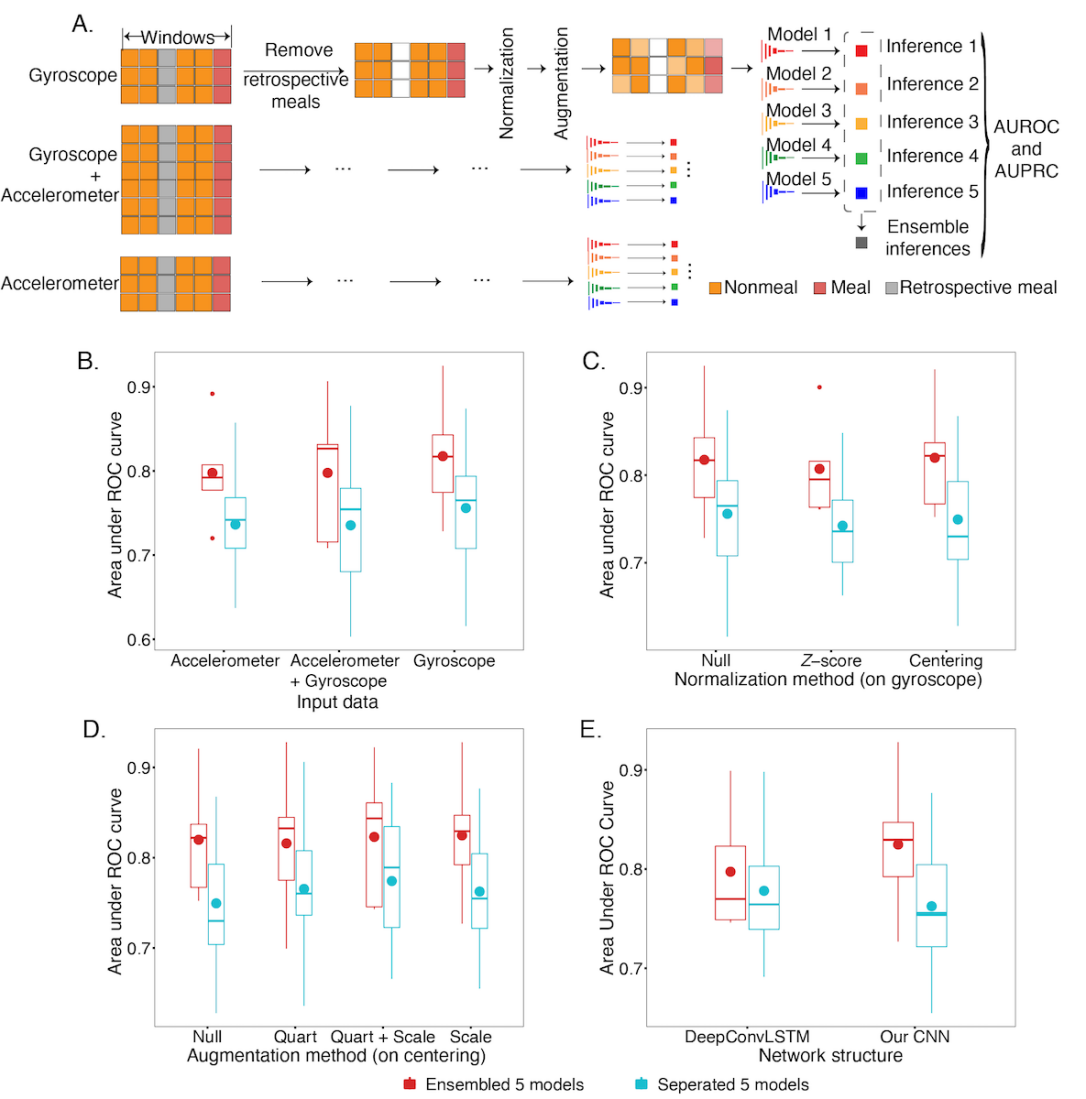
Evaluation of model performance on 5-minute windows of the discovery cohort. (A) Models were built by gyroscope data only, accelerometer data only, and gyroscope+accelerometer data. Next, we tested the centering and normalization of each axis of the data. Intensive data augmentation was applied to the data on the fly. For each method, 5 models were trained by resampling the training and validation data and they were assembled for evaluation. (B) Presents the performance comparisons of different data selections. (C) Presents the performance comparison of different normalization methods applied on the gyroscope model. (D) Presents the performance comparisons of the augmentation methods based on the centering model, where Quart refers to the quaternion rotation augmentation, and Scale refers to scaling the magnitude. (E) Comparison of the performances between DeepConvLSTM and the method presented in this paper. AUROC: area under the ROC curve; AUPRC: area under the precision–recall curve; CNN: convolutional neural network; ROC: receiver operator characteristic.

**Figure 4:**
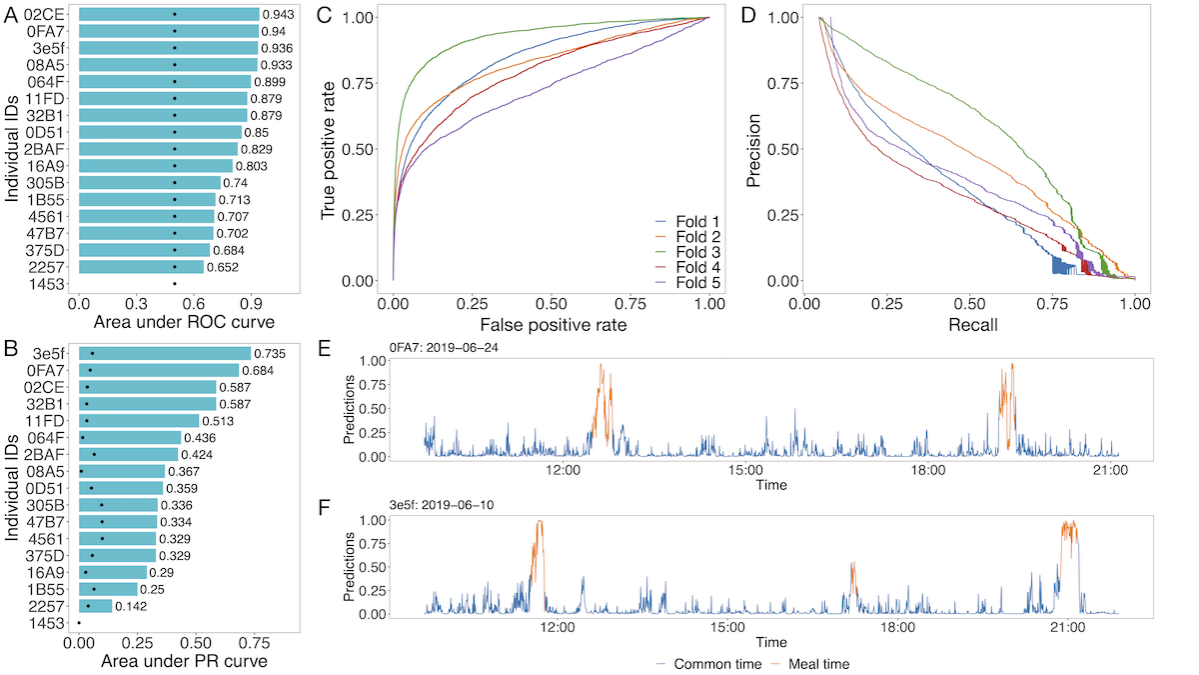
The evaluations of the selected best model: convolutional neural network backbone trained with the centering normalized gyroscope data using magnitude scaling. (A and B) show the leave-one-subject-out results of the model on the discovery cohort, evaluated both in the area under the curves and area under the precision–recall curves. The black points in (B) are the baselines for the individuals. As 1453 does not have any positive samples after excluding the retrospectives, its value will be empty. (C) is an area under the ROC curve, and (D) is a precision–recall curve for the cross-validation from the ensemble model, respectively. (E and F) give the inferences of the 2 dates of records, where the blue segments denote the signals of the non–meal time, and the orange segments are the signals of the mealtime. PR: precision recall; ROC: receiver operator characteristic.

With the generated 5-minute windows (Figure 1D), we constructed a 1D (along the time axis for both input and output) deep learning model (Figure 1F) with 3 channels as input (x, y, and z axes of accelerometer or gyroscope; 6 channels when giving both accelerometer and gyroscope information). On the basis of the cross-validation described in the *Methods* section, our work showed an average AUC of 0.825 (SD 0.073; Figure 3D and Figure 4C) and an average AUPRC of 0.437 (SD 0.096), with the baseline (same value predictions for all data points) of 0.053 (Multimedia Appendix 2, Figure S3C and Figure 4D). When including the retrospective meals in prediction, our model showed stable performances with an average AUC of 0.813 (SD 0.067) and AUPRC of 0.440 (SD 0.077, baseline 0.065). In comparison, we adapted DeepConvLSTM on this data set [22], which achieved an average AUC of 0.797 (SD 0.065) and an average AUPRC of 0.294 (SD 0.072; Figure 3E) on the nonretrospective meals. This demonstrated that the techniques integrated into this approach could substantially improve over a state-of-the-field method.

We identified the factors that affect performance. First, based on the 5 models trained on the random splits of the training set, assembling the inference values from the output of the last fully connected layer, by taking the averages in each test, can significantly improve the performances in all the experiments (P<.001; Figure 3B-Figure 3D; Multimedia Appendix 2, Figure S3A-3C). Second, building the model on gyroscope data can achieve better performances than using accelerometer data or both. The average AUC and AUPRC of the gyroscope model are 0.02 to 0.05 higher than the other alternatives (P values for AUCs <.001; P values for AUPRCs <.001; Figure 3B and Multimedia Appendix 2, Figure S3A; Multimedia Appendix 2, Tables S1 and S2). Third, choosing correct input data normalization methods may be helpful. Centering normalization improved the model performance by 0.002 on the AUC and 0.01 on the AUPRC (P values for AUCs=.29; P values for AUPRCs=.10), whereas, with the *z* score normalization, which may compress the original ranges of the signals, the performances will drop by 0.01 and 0.04 on the AUC and AUPRC (Figure 3C and Multimedia Appendix 2, S3B; Multimedia Appendix 2, Tables S3 and S4). This is likely to reflect the fact that the magnitude of the signal is critical to the model, whereas the directions of the watch (ie, reflected as the overall shift of an axis) are not relevant. Fourth, data augmentation, including the quaternion rotation of the signals in space and scaling the signal magnitudes, might improve the model performance. Rotation and scaling can provide >0.01 improvement on both AUCs and AUPRCs for a single model, although not statistically significant (P values for AUCs=.33; P values for AUPRCs=.17). When considering the ensemble model that aggregates 5 models generated using different random seeds, the magnitude scaling gives better but not significantly better performance both on AUC and AUPRC (P values for AUCs=.26; P values for AUPRCs=.88; Figure 3D and Multimedia Appendix 2, Figure S3C; Multimedia Appendix 2, Tables S3 and S4). Adding in local time did not improve the performance (Tables S1 and S2).

To retrieve the performances of each individual, including the previously excluded ones, and generate the baselines for evaluating the improvements of our following fine-tunings on the personalized models, we also used the leave-one-subject-out approach to calculate the AUCs and AUPRCs. For each individual, the model was trained on all the other data except the one left out. The average AUC for the ensemble model was 0.818 (SD 0.104), and the average AUPRC was 0.419 (SD 0.162, Figure 4A and Figure 4B; Multimedia Appendix 2, Table S7). Visualization of the inferences of two dates of records: data on June 24, 2019, from 0FA7 and data on June 10, 2019, from 3E5F show consistency with the eating and noneating behaviors (Figure 4E and Figure 4F).

### Fine-tuning of the Personalized Model Improves Performance

**Figure 5:**
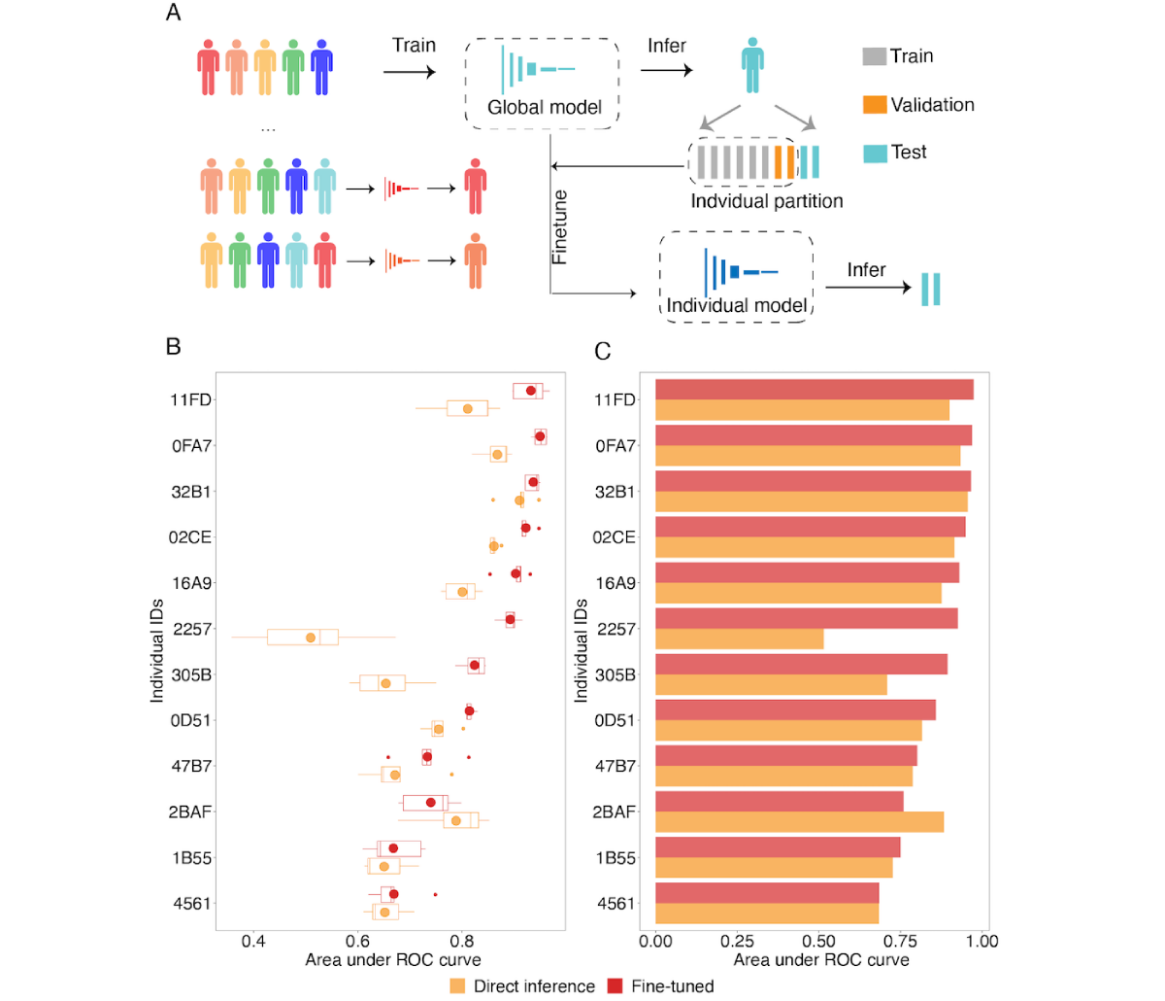
Evaluating fine-tuning to generate individual models on the discovery cohort. (A) For a specific individual under investigation, we first trained 5 global models using all other individuals by resampling the training and validation set for the deep learning training process. Next, we split records of the individual of interest by days into training, validation, and test sets and fine-tuned the global model using the training and validation set. We evaluated the performance by the area under the ROC curve for both the global and the individual fine-tuned models for (B) 5 separated models and (C) ensemble models. ROC: receiver operator characteristic.

This longitudinal data allowed us to explore whether it is possible to construct personalized models for eating and further improve model performance (Figure 5A). The global models served as the fine-tuning starting points for the individuals of interest (Figure 4A and Figure 4B). This study design mimics an important utility of the models in real life, where we adopted an existing model to a previously unseen person and asked whether we could improve the inference on this individual by observing some data for this individual.

Comparing the performance of the global model on this individual versus the fine-tuned model, we found that other than 1 individual (2BAF), the fine-tuned personalized models showed better performance than directly applying the population models on the specific individuals. The AUC on average improved for the fine-tuning model to 0.872 (SD 0.099), with an average weighted F1 (the average weights were 0.059 and 0.941 for positives and negatives, respectively) score of 0.938 (SD 0.048), an average precision of 0.945 (SD 0.045), and an average recall of 0.934 (SD 0.049; Figure 5B and Figure 5C; Multimedia Appendix 2, Tables S8 and S9).

### Aggregation of Multiple Windows Reaches Near-Perfect Detection of Meal Events

**Figure 6:**
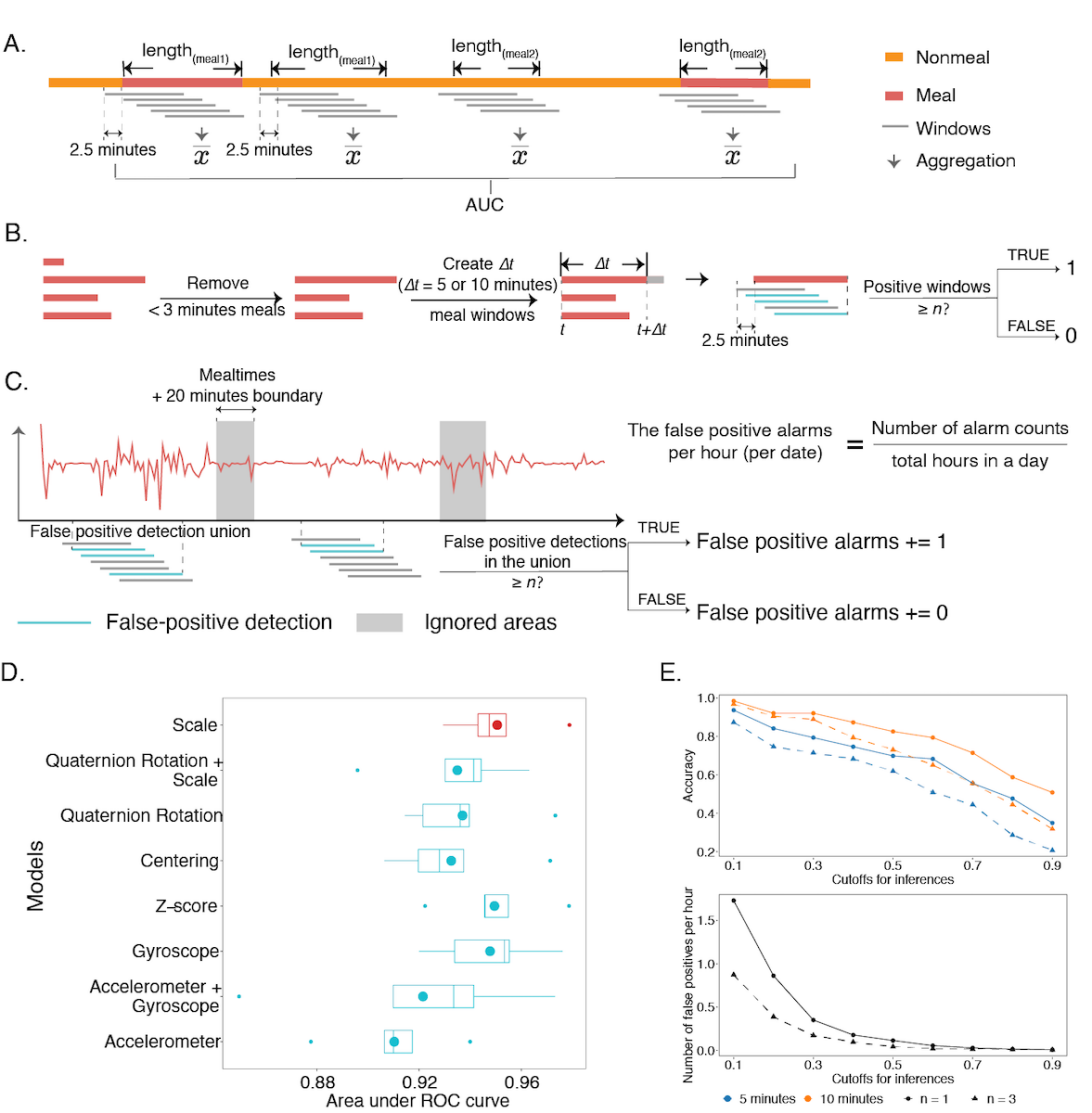
The results of the aggregations on the entire meals and on the specific time regions after meal start. (A) Aggregation to produce meal-level detection performance. (B) A preprocessing step removed meals that were <3 minutes and created 5- or 10-minute windows for positive examples to evaluate recall rate. (C) Nonmeal regions were used to calculate false positive alarms using the 5-minute windows and the same criteria as what was used to define positive inferences in calculating the recall rate. The gray areas denote the mealtimes with the 10-minute boundaries at the start and the end, where the windows are out of consideration. (S) Shows the evaluations of the aggregations on the entire meals. The boxplots comprise the AUCs from the average inferred scores of the ensemble models in the cross-validation, and the experiments (models) are the same as those in Figure 3A. The point in each box denotes the corresponding average AUC. (E) indicates the results of the aggregation on the 5- and 10-minute meals after starting and on the entire negative signals. The lines show how the detection accuracy and the hourly false positive numbers (the black lines) change along with the cutoffs. The orange lines show the results for the 10-minute meals, and the blue lines are the 5-minute meals. The shape of the points represents the choice of N, where the circles/solid lines are N=1, and the triangles/dashed lines are N=3. AUC: area under the curve; ROC: receiver operator characteristic.

We then evaluated the model performance on the original mealtimes. We conducted three experiments on cross-validation of the discovery cohort:(1) the prediction for whole meals (Figure 6A), (2) the prediction within 5 minutes or 10 minutes after the meal starts (Figure 6B), and (3) the false calls within an hour (Figure 6C). For all nonretrospectively recorded meal events, we calculated the average score during each meal event. For calculating scores for the negative regions, we randomly selected a series of negative regions whose lengths and numbers were matched to the meal events. The scores for the negatives were generated by taking the averages of the windows within the selected regions. The models achieved an AUC of 0.951 (SD 0.018) by this aggregation, and the corresponding weighted F1 score (weights were 0.464 and 0.536 for positives and negatives, respectively), precision, and recall were 0.877 (SD 0.037), 0.8890 (SD 0.027), and 0.879 (SD 0.035), respectively (Figure 6D; Multimedia Appendix 2, Table S10). Including the retrospective, meals would result in a similar AUC of 0.951 (SD 0.017), with the corresponding weighted F1 score of 0.858 (SD 0.040, weights for positives and negatives were 0.5).

For prediction on the 10-minute or 5-minute after meal start, we used the accuracy (ie, how many mealtimes were correctly inferred) for evaluation (Figure 6E; Multimedia Appendix 2, Tables S11 and S12). In this case, we were interested in how we could choose a criterion so that most meals could be detected within 5/10 minutes. We used a moving step of 10 seconds and defined a window to be positive if the prediction score was >0.3. If >3 windows were predicted to be positive in 5/10 minutes, we alarmed a *call*. Using these criteria, we reached a recall rate of 0.889 for 10 minutes, and the result remained robust in the 5-minute test, cutoffs of prediction score between 0.4 and 0.6, and the number of windows we used to alarm the call.

Next, we calculated the number of false positive predictions per hour of negative regions. For each hour, we had a total of 360 chunks (with a moving step of 10 seconds). The corresponding false positive prediction was 0.172 per hour; that is, 1 to 2 times of false positives in a whole day of activity. Again, this result was robust against the cutoff defining a positive window and the number of windows needed to alarm a call.

### Generalizability to an Independent Validation Cohort Collected in a Different Season

Although the first batch of the data was collected in the summer, we proceeded to collect a second validation cohort in winter, 6 months later, by recruiting 17 new individuals. By splitting the discovery cohort data into 5 sets of training and validation data, we first finalized 5 models for the first cohort; then, we directly applied these models to the validation cohort for inferences. Next, we applied the scheme of the whole meal predictions to the validation cohort, both on the data with and without the retrospective meals. Without any further tuning, the model achieved a meal-level AUC of 0.941 on the validation cohort for the nonretrospective meals, with a 0.870 weighted F1 score (the weights were 0.445 and 0.555 for positives and negatives, respectively), a 0.878 precision, and a 0.871 recall. With the retrospective meals, the meal-level AUC and the weighted F1 score were 0.920 and 0.846, respectively (the weights for positives and negatives were 0.5). The performances of our work in this study are listed in Table 2. 

**Table 2:** List of the performances in this study.

Experiments	Area under the curve	Area under the precision–recall curve	Weighted F1 score
Cross-validation of our model on 5-minute windows	0.825	0.437	N/A^a^
Cross-validation of our model on 5-minute windows, including the predictions on retrospective meals	0.813	0.440	N/A
Cross-validation of DeepConvLSTM [22] on 5-minute windows	0.797	0.294	N/A
Leave-one-subject-out approach of our best model on 5-minute windows	0.818	0.419	N/A
Fine-tuning the personalized model	0.872	N/A	0.938
Cross-validation of our model on the original mealtimes (discovery cohort)	0.951	N/A	0.877
Cross-validation of our model on the original mealtimes, including the predictions of retrospective meals (discovery cohort)	0.951	N/A	0.858
Predictions of our model on the original mealtimes (validation cohort)	0.941	N/A	0.870
Predictions of our model on the original mealtimes, including the predictions of retrospective meals (validation cohort)	0.920	N/A	0.846
Accuracy of detecting the eating in 10 minutes	0.889	N/A	N/A
False positive detections per hour	0.172	N/A	N/A

^a^N/A: not applicable.

## Discussion

### Principal Findings

In this study, we presented a large, in-the-field, digital eating detection study of eating activity. Deep learning algorithms experimented with a diverse array of augmentation, preprocessing, and architectures allowed us to narrow down the algorithm into one with a performance of AUC of 0.825 to infer previously unseen individuals for a single 5-minute window. When evaluated on the entire meal regions, this AUC was 0.951. We further validated the algorithm in an independently time-lapsed cohort collected in a different season (6 months later, winter) and achieved a meal-level performance of 0.941 AUC without further tuning. This design can potentially result in models that are more or at least similarly generalizable than data collected consecutively in the same season. This represents the first study that harbors a validation cohort in this field.

Compared with other studies that focus on population-wise models [9,13,24-26], the longitudinal weeks of follow-ups of the data set presented in this study allowed us to further explore the possibility of personalized models for detecting eating activity. It is widely recognized that eating motions differ substantially in a population by gender, culture, and certainly individual habits. This fine-tuning scheme produced an average AUC at 0.872, corresponding to a 0.89 success rate in calling back an eating event within 10 minutes. This substantial improvement in performance points to the direction toward personalized eating monitoring in the dietary research field.

Records of the local time, as well as the utensils used for each meal, also allow us to glean insight into their influences on our model (Multimedia Appendix 2, Figure S4). We found that food taken with hands had relatively poor performance (AUC=0.812; Multimedia Appendix 2, Figure S4B). In addition, we found that false positive rates are relatively high between 6 AM to 7 AM and 9 PM, indicating potential morning and evening activities mimicking eating movement (Multimedia Appendix 2, Figure S4C). Future studies incorporating different characteristics of utensils as well as whole daily activity logs might have the potential to further improve the performance.

### Limitations

We acknowledge several potential limitations of this study. First, we excluded smoking individuals, for whom the inference task could become more complicated as the motion of smoking shares a certain similarity with the motion of eating. Second, we only included healthy individuals, which may not be representative of the population of movement disorders such as ataxia and Parkinson disease. In addition, we did not collect the data for the nondominant hand. The weaker and noisy signals may significantly affect our model built on dominant hand data. Potentially, combined with additional devices such as ear- and chest-anchored devices and video (Multimedia Appendix 2, Table S15 [27-31]), in future works, we will be able to combat such limitations. We used a total of 34 individuals in the study. Although we observed strong predictions across individuals, larger collections focusing on more individuals but less longitudinal follow-up might further complement the information provided in this study. Furthermore, we used 50 Hz data in this study for optimizing battery performance in collecting data. It is yet to be evaluated how higher Hz data contribute to performance with the development of the devices.

### Future Works and Conclusions

This study and the API developed here open several future directions that are worth exploration. For example, how do digital indicators differ for populations coming from different cultural backgrounds? Does handedness affect model construction and performance? And how much will the model be affected if one wears the device on his or her nondominant hand? Answering these questions will need large-scale studies with a large number of participants, and the API and data streaming platform developed in this study will become a convenient tool for this purpose. The accuracy of the models developed in this study satisfies immediate deployment needs in clinical settings to monitor eating behavior and give guidance to treatment regimen adjustment accordingly. We envision the digital streaming platform will be widely integrated into a variety of clinical trials in the near future.

## Appendix

Multimedia Appendix 1 The study consent form.

Multimedia Appendix 2 The supplementary tables and figures.

Multimedia Appendix 3 The codes for our models and experiments in this study.

## Abbreviations

API: application programming interface
AUC: area under the curve
AUPRC: area under the precision–recall curve
