# Supplementary material for: Enabling Eating Detection in a Free-living Environment: Integrative Engineering and Machine Learning Study
Source: J Med Internet Res. 2022 Mar 1;24(3):e27934. doi: 10.2196/27934 (PMC8924783; doi:10.2196/27934)
Supplement: Multimedia Appendix 1 [file jmir_v24i3e27934_app1.doc]

Eli Lilly and Company, Indianapolis, Indiana USA 46285

**Informed Consent Form**

| Name of Study: | Prospective, Non-interventional, Proof-of-Concept Study to Enable Development of Pattern Recognition Algorithms for Detecting Periods of Food Intake Using Passively Collected Data from Wearable Device Motion Sensors |
| --- | --- |
| Study Number: | 2019-8193  WIRB® Protocol #20190878 |
| Eli Lilly Investigator: | Bohdana Ratitch, PhD  Principal Research Scientist  Advanced Analytics and Data Sciences: Machine Learning, Artificial Intelligence, and Connected Care  Eli Lilly and Company  507 Grosvenor Ave  Westmount, Quebec H3Y2S5  Canada |
| Study Sponsor: | Eli Lilly and Company  Lilly Corporate Center  Indianapolis, IN 46285 |
| Study-Related  Phone Number(s) | 1-317-842-7834 (24 hours) |

**This Informed Consent Form has two parts:**

1. Information Sheet (information about the study)
2. Certificate of Consent (for signatures if you choose to participate)

**Voluntary Participation**

Please be assured that your participation in this study is entirely voluntary. Your decision to participate or not, your compliance with the study procedures should you decide to participate, or your decision to withdraw from the study at any time will not impact your employment or your performance evaluation.

**You will be given a copy of the full Informed Consent Form.**

## Part I: Information Sheet

**Purpose of this study**

The purpose of this study is to develop and evaluate a set of pattern recognition algorithms that could automatically detect periods of food intake (when a person is eating or drinking) based on passive monitoring of motion sensor data from wearable devices in everyday living conditions. The study is designed to collect data required to pursue the analytical research. The study motivation is that an automatic detection of food intake can improve performance of connected care diabetes devices developed by Eli Lilly. This is a feasibility study to inform future research on the use of passively collected data in connected care.

**What you are being asked to do in this study**

You are being asked to wear a smartwatch, Apple Watch Series 4, which will be provided to you for the duration of your participation in the study, up to 14 days. This smartwatch is equipped with motion sensors (accelerometer and gyroscope) and the measurements from these sensors will be continuously collected as you go about your normal daily activities. You are being asked to wear the Apple Watch on the arm that you consider dominant for eating. You are also being asked to use an application installed on the Apple Watch to log the times during which you consume food or beverages (start and stop time) and the type of utensils you use, for the duration of the study. You will also need to use your Lilly iPhone to set up and connect to Apple Watch and to use an iPhone Research App developed for this study to facilitate study data flow. More details are provided below in the “Study procedures” section.

**Study procedures**

If you agree to participate in this study, your participation will involve wearing an Apple Watch on your wrist. The smartwatch will provide the study team with data about the movements of your arm as you go about your normal daily activities. This data will be used to develop machine learning algorithms to automatically detect when an individual is eating or drinking based on the movements of their arm.

Your participation in the study will involve the following.

- You will be assigned a numeric Participant ID at the beginning of the study. All data collected during the study, regardless of method of collection, will only be linked to this Participant ID, and will not be linked to your name or contact information.
- We will provide you with an Apple Watch Series 4, and we will assist you with the setup of the device. The Apple Watch will be paired to your Lilly iPhone. To receive the Apple Watch and study-related instructions you will have a choice between attending a group deployment meeting with other study participants or meeting with the study coordinator individually. At the group deployment meeting, a project team member will go over the study-related instructions, assist participants with the setup of the device and the Research App, and will be available to answer participants’ questions. If you prefer to meet with the study coordinator individually, the device and instructions will be provided to you by the study coordinator and arrangements will be made to provide you technical assistance with the set-up should you need it.
- You will be asked to wear the Apple Watch on the wrist of the arm that you consider dominant for eating for the study duration, which is 14 days.
- You will be asked to wear the Apple Watch during day-time, i.e., to put it on your wrist after you wake up and to remove it before you go to bed. We understand that it may not be feasible to wear the Apple Watch at all times during the day. When not possible or desirable to wear it (e.g., during showering), you may take it off. You will recharge your Apple Watch during night-time. You are not being asked to change your daily activities in any way.
- An application developed for this study will be pre-installed on your Apple Watch. It will continuously record the measurements from the device’s two motion sensors: accelerometer and gyroscope. This data will be collected passively, i.e., not requiring any active input on your part, except to start the recording in the morning and end the recording at the end of the day.
- You will be asked to log periods when you consume any food or beverages (e.g., meals, snacks, coffee) if you estimate that it will involve more than three bites or sips and last for more than two minutes. You will use an application installed on your Apple Watch to log the following information: the start time of your food intake (by tapping a “Start” button on the Apple Watch application), the end time of your food intake (by tapping an “End” button on the Apple Watch application), and type of utensils used (by selecting applicable check-boxes such on the Apple Watch application). If you forget to log a period of food intake on the Apple Watch, you will be able to retrospectively enter approximate times of your meal using a study application installed on your iPhone.
- You will be asked to complete a two-item questionnaire to assess your perceived burden of wearing the Apple Watch and completing food intake logs (on a scale of 0 to 10) 4 times during the study using the study application installed on your iPhone.
- You will be asked to provide some additional information, which will be collected through a secure online form. This information will include: the arm you consider dominant for eating activities (left or right), your age group (less than or equal to 35 years old or greater than 35 years old), gender, race category (“Caucasian, Black or African American”, versus “Asian, Hispanic, or Other”), and whether you have prior experience using an Apple Watch.
- Data collected throughout the study as described above will be transferred using an encrypted data transfer method and stored in a secure limited-access location designated for this study, with similar protections and company policies as used for other Eli Lilly confidential data. You will use the study app on your iPhone to trigger data upload at the end of each day.
- You will return the Apple Watch provided to you at the end of the study, that is after 14 days or earlier, should you decide to withdraw from the study sooner. You will reset the Apple Watch to its factory settings before returning it, which will ensure no further collection of data from the device.

**Study duration and estimated time commitments**

You are being asked to participate in this study for 14 days.

If you decide to take part in the study, you will meet with a project team member twice: at the beginning of the study (a session of up to 30 minutes) and at the end of the study (a session of up to 15 minutes). You will need to spend a few minutes of your time each day to handle the Apple Watch, recharge it, and to log the periods when you consume food. You will also need to spend up to 5 minutes to complete a two-item questionnaire four times during the study.

**Risks or Discomforts**

- Wearing the Apple Watch on the wrist of your dominant arm might be uncomfortable.
- You may find it bothersome to log the periods of time when you consume food or beverages.
- Wearable devices may contribute to distraction when operating a motor vehicle. We ask that you do not handle or consult the Apple Watch while driving, regardless of whether you consume any food or beverage during that time.
- You may be concerned about your privacy because of collection of data from Apple Watch. We will be collecting data only from motion sensors that may tell us about the movements of your arm in relation to the periods of food intake that you will log.
- You may be concerned about the security of the data collected during the study. We strive to handle all data collected in this study with the same level of protections as any personal and confidential data within Eli Lilly systems.
- You may be concerned about the impact your participation in the study or decision not to participate may have on your employment with Eli Lilly. We assure you that it will not bear any effect on your employment or performance evaluation. If you have questions about your rights as a research subject or if you have questions, concerns, or complaints about the research, you may contact:

Western Institutional Review Board® (WIRB®)

1019 39th Avenue SE Suite 120

Puyallup, Washington 98374-2115

Telephone: 1-800-562-4789 or 360-252-2500

E-mail: Help@wirb.com

WIRB is a group of people who perform independent review of research.

**Benefits**

You will likely not derive any direct benefit from this study. However, society and investigators will benefit from the knowledge gained if the capability of accurately detecting periods of food intake from passive motion sensor data is confirmed and refined. This study is expected to provide benefits to Eli Lilly in terms of developing core capabilities and expertise in promising areas of digital health for improving patient care.

**Reimbursements**

Participants will not be reimbursed from this study.

**Costs to You**

There are no costs to you, other than your time, for participating in this study.

If the watch is damaged during the study, participants are expected to return the damaged watch to study investigator without cost to the participants. In addition, should you lose your Apple Watch or have other issues with its operation, you must notify the study coordinator, Christopher Brian Wittman by email at [wittman_christopher@network.lilly.com](mailto:wittman_christopher@network.lilly.com) or phone 317-842-7834, as soon as possible for appropriate follow-up.

**Alternatives**

Your alternative is not to participate.

**Confidentiality and Data Use**

As part of the conduct of this research study, it will be necessary to share personal information about you with the project team. This Informed Consent Form explains how your personal information will be used and to whom it will be given (“disclosed”) for this research study. It also describes your privacy rights, according to a federal government rule that has been issued to protect the privacy rights of research subjects. This rule was issued under a law called the Health Insurance Portability and Accountability Act of 1996 (HIPAA). This rule is designed to protect the confidentiality of your personal information.

Your personal information is information about you that could be used to find out who you are. For this research study, this includes information created or collected during the study.

By signing the Informed Consent Form for this study, you give permission (“authorization”) for the uses and disclosures of your personal information that are described in this Informed Consent Form. If you do not want to allow these uses, you should not participate in this study.

If you agree to participate in the research study, your personal health information will be used and disclosed in the following ways:

- You will be assigned a numeric Participant ID to identify all digital data you generate. All collected study data will be linked to this Participant ID and will not be linked to your contact information such as name, address, phone number, e-mail, Lilly ID, etc.
- All digital data will belong to Eli Lilly and we strive to secure it in the same way as we treat other confidential data.
- The project team from the Eli Lilly Advanced Analytics and Data Sciences group (“the sponsor”) will access your study-related data (“study data”) for research purposes to support the scientific objectives of the study described in the consent document or to improve the design of future studies.
- Because the sponsor conducts business related to clinical research in many countries around the world, this may involve sending your study data outside of this country.
- Your study data, either alone or combined with data from other studies, may be shared with regulatory authorities in this country (such as the FDA) and other countries including the United States and also with the ethics review board overseeing this study.
- Study data that does not identify you may be published in medical journals or shared with others as part of scientific discussions.
- The sponsor works with business partners in drug development. The sponsor may share your study data with these business partners, but only if the business partners need the information as a part of their work with the sponsor, and only if the business partners sign a contract that requires them to protect your study data in the same way as the sponsor.

You have the right to see and copy your personal information related to the research study for as long as this information is held by Eli Lilly as needed for legitimate business purposes according to Eli Lilly’s record retention policies and applicable laws and regulations.

You may cancel your authorization at any time by providing a written notice to the study coordinator, Christopher Brian Wittman by email at [wittman_christopher@network.lilly.com](mailto:wittman_christopher@network.lilly.com). If you cancel your authorization, the study health care provider and staff will no longer use or disclose your personal information in connection with this study, unless the study staff needs to use or disclose some of your personal information to preserve the scientific integrity of the study. The sponsor will still use study data that was collected before you canceled your authorization. If you cancel your authorization, you will no longer be able to participate in the study. However, if you decide to cancel your authorization and withdraw from the study, or decide not to provide your authorization and not participate in the study, you will not be penalized or lose any benefits to which you are otherwise entitled.

Your authorization for the uses and disclosures described in this Informed Consent Form does not have an expiration date.

**This proposal has been reviewed by Eli Lilly and Company, which is sponsoring the study. It has also been reviewed by Western Institutional Review Board, which is a group of people who perform independent review of research whose task it is to make sure that research participants are protected from harm. If you wish to find about more about the IRB or if you have questions about your rights as a research subject or if you have questions, concerns, or complaints about the research, you may contact:**

**Western Institutional Review Board® (WIRB®)**

**1019 39th Avenue SE Suite 120**

**Puyallup, Washington 98374-2115**

**Telephone: 1-800-562-4789 or 360-252-2500**

**E-mail: Help@wirb.com**

If you have questions, concerns, or complaints, or think this research has hurt you or made you sick, talk to the research team at the phone number listed above on the first page.

**Part II: Certificate of Consent**

To become a part of this study and to authorize use and disclosure of your personal information, you must sign and date this page.

By signing this page, you are confirming the following:

- You have read all of the information in this Informed Consent Form, and you have had time to think about it.
- All of your questions have been answered to your satisfaction.
- You are volunteering to be part of this research study, and you understand that you may freely choose to stop being a part of this study at any time.
- You allow the study team to use and disclose your personal information as described in this document.
- You have received a copy of this Informed Consent Form to keep for yourself.

Signature of study participant Date (dd-mmm-yyyy)

(Study Participant must personally date)

Study participant name (print or type) Study Participant Initials and Participant ID

Name of individual conducting consent

discussion (print or type)

Signature of individual conducting consent Date (dd-mmm-yyyy)

discussion (Individual conducting consent discussion

must personally date)
