# Supplementary material for: Enabling Eating Detection in a Free-living Environment: Integrative Engineering and Machine Learning Study
Source: J Med Internet Res. 2022 Mar 1;24(3):e27934. doi: 10.2196/27934 (PMC8924783; doi:10.2196/27934)
Supplement: Multimedia Appendix 2 [file jmir_v24i3e27934_app2.pdf]

Supplementary materials for:

Zhang *et al., Enabling Eating Detection in a Free-living Environment*

Figure S1: Summary of the sampling information for training and validation cohort. (a) shows the distribution of the sample lengths in hours, and (b, c) shows the distribution of the sample start time and end time.

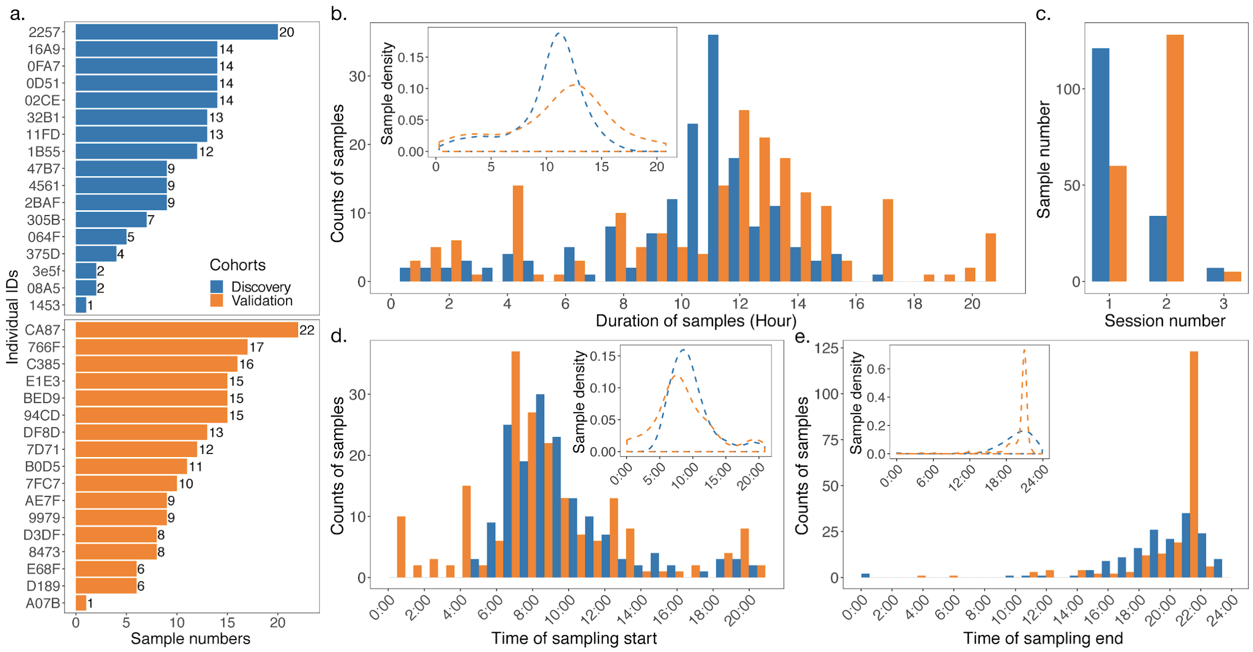

Figure S2: Two examples of the problematical datasets, the blue lines denotes the meal time, and the grey lines denotes the common signals. (a) corresponds to the short sampling time problem, where the sampling just starts and ends before and after the meal; (b) shows the weak signal problem. There is a plain line in the signal, which was not recorded as an interruption.

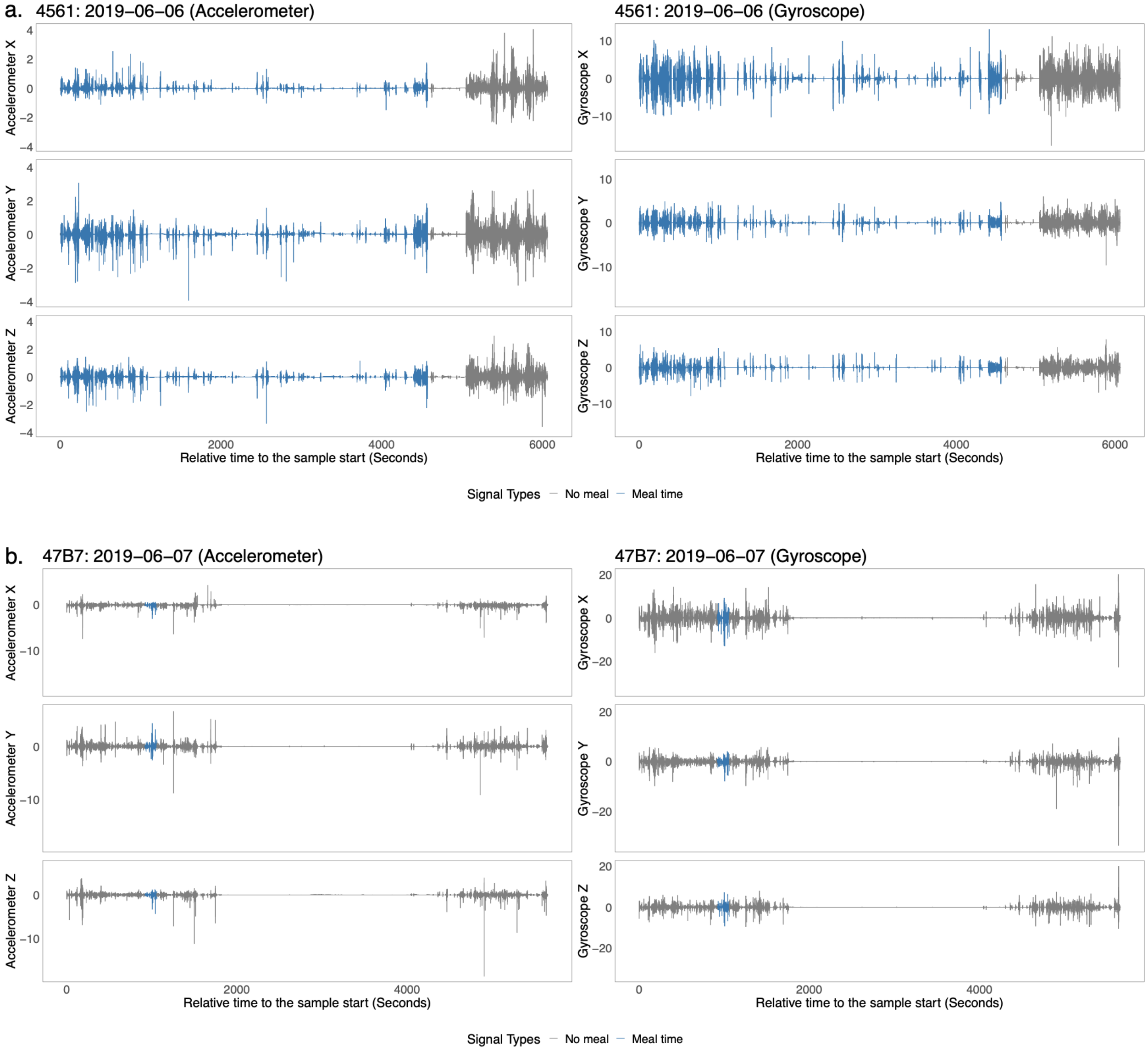

Figure S3: AUPRC results of the experiments. (a) is the experiment of data selection. (b) presents the experiments of choosing a normalization method based on the gyroscope model, and (c) shows the experiments of the augmentation methods based on the centering model.

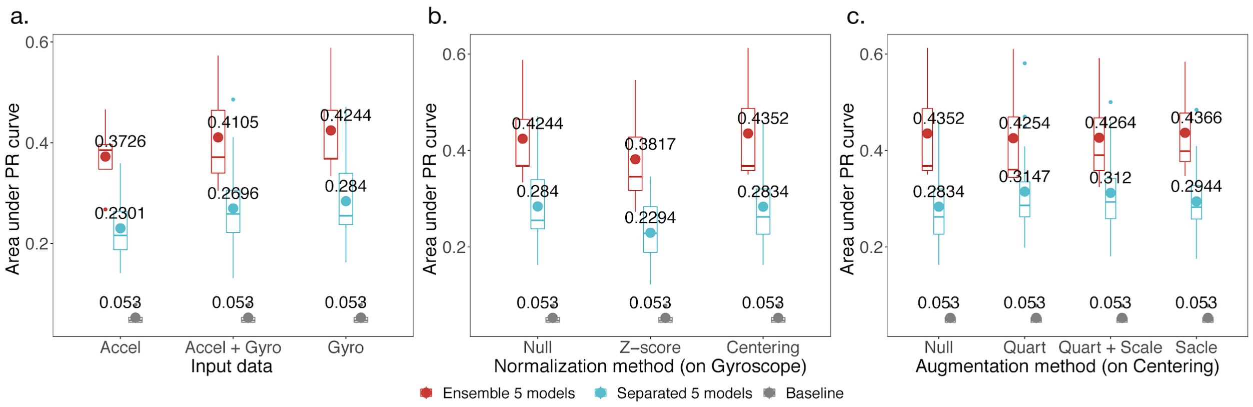

Figure S4: The effects of the utensils on the model predictions and performances. (a, b) show the correlations on the utensils, where (a) is the prediction scores and (b) is the AUCs computing by combining the meal records with randomly sampling negative records, whose size is three times larger. (c) is the average prediction scores (the red points and line) and the false positive rates (the blue bars) changing along with the meal times.

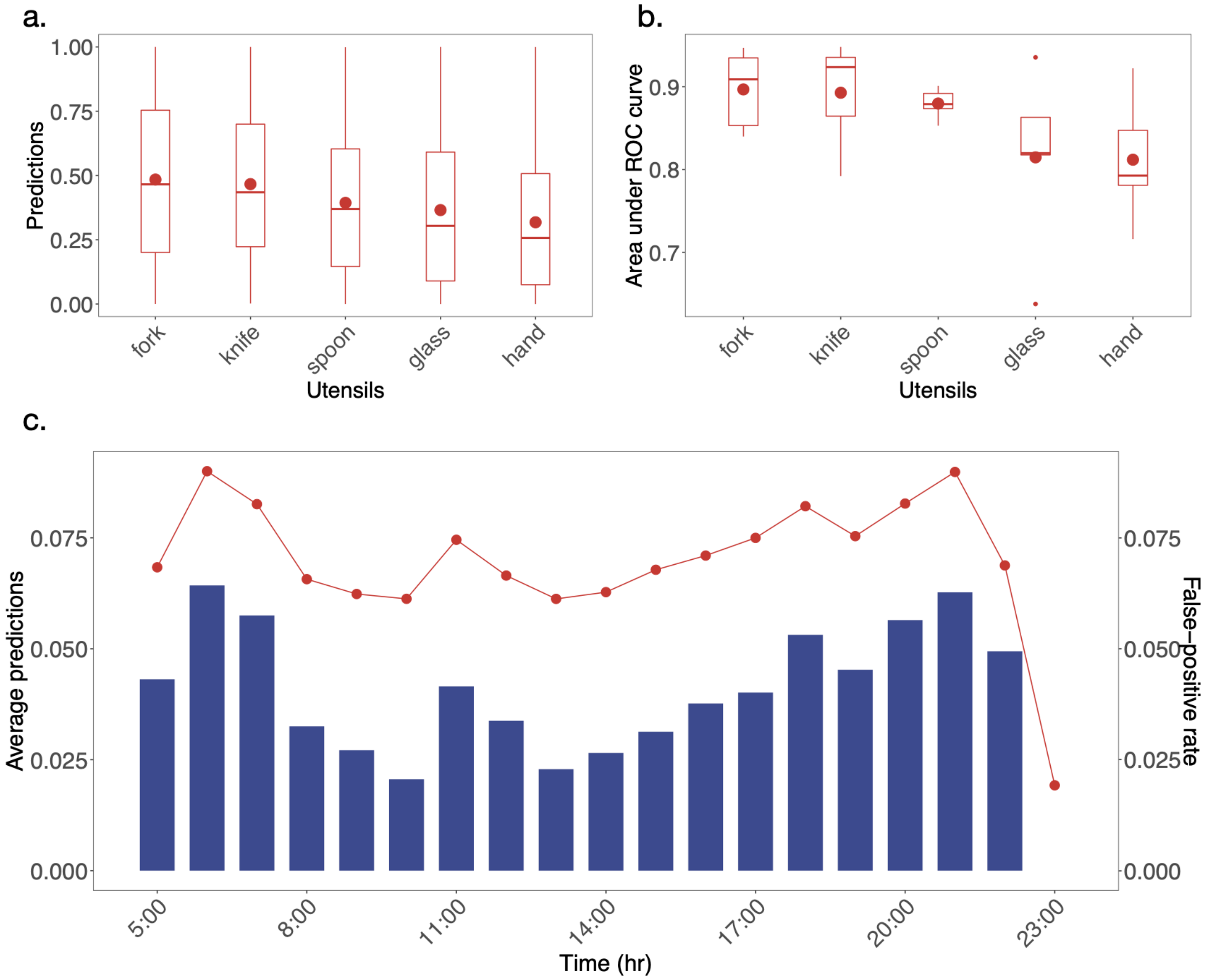

Table S1. AUCs for the experiments on data selection

Data	Min.	Max.	Mean	Median
Accelerometer	0.7201	0.8918	0.7977	0.7921
Gyroscope	0.7283	0.9250	0.8175	0.8170
Gyroscope + local time	0.7150	0.9139	0.8114	0.8150
Accelerometer + Gyroscope	0.7082	0.9068	0.7977	0.8266

Table S2. AUPRCs for the experiments on data selection

Data	Min.	Max.	Mean	Median	Baseline (mean)
Accelerometer	0.2676	0.4660	0.3725	0.3854	0.0530
Gyroscope	0.3339	0.5879	0.4244	0.3685	0.0530
Gyroscope + local time	0.3350	0.5496	0.4008	0.3483	0.0530
Accelerometer + Gyroscope	0.3047	0.5725	0.4105	0.3712	0.0530

Table S3. AUCs for the experiments on normalization method

Normalization methods (On gyroscope model)	Min.	Max.	Mean	Median
Z-score	0.7611	0.9005	0.8072	0.7952
Centering	0.7523	0.9208	0.8199	0.8221

Table S4. AUPRCs for the experiments on normalization method

Normalization methods (On gyroscope model)	Min.	Max.	Mean	Median	Baseline (mean)
Z-score	0.2727	0.5459	0.3817	0.3456	0.0530

Centering	0.3502	0.6124	0.4352	0.3680	0.0530

Table S5. AUCs for the experiments on augmentations

Augmentations (On centering model)	Min.	Max.	Mean	Median
Quaternion rotation	0.6992	0.9281	0.8159	0.8325
Magnitude scaling	0.7270	0.9278	0.8247	0.8294
Quaternion rotation + Magnitude scaling	0.7431	0.9223	0.8230	0.8435

Table S6. AUPRCs for the experiments on augmentations

Augmentations (On centering model)	Min.	Max.	Mean	Median	Baseline (mean)
Quaternion rotation	0.3426	0.6103	0.4254	0.3602	0.0530
Magnitude scaling	0.3467	0.5837	0.4366	0.3983	0.0530
Quaternion rotation + Magnitude scaling	0.3244	0.5912	0.4264	0.3904	0.0530

Table S7. AUCs and AUPRCs for each individual in the population models

AUC		AUPRC		
**Patient**	**Ensemble model**	**Patient**	**Ensemble model**	**Baseline**
02CE	0.94279	02CE	0.58668	0.03534
064F	0.89854	064F	0.43632	0.01557
08A5	0.93263	08A5	0.36747	0.00966
0D51	0.85046	0D51	0.35915	0.05315
0FA7	0.9397	0FA7	0.68408	0.04786
11FD	0.87925	11FD	0.51347	0.03341
16A9	0.80284	16A9	0.29021	0.02848

1B55	0.7133	1B55	0.24996	0.06408
2257	0.6516	2257	0.14184	0.03935
2BAF	0.82917	2BAF	0.4235	0.06505
305B	0.74037	305B	0.33642	0.09673
32B1	0.87919	32B1	0.58658	0.03275
375D	0.68413	375D	0.3287	0.05651
3e5f	0.93554	3e5f	0.73509	0.05738
4561	0.7066	4561	0.32906	0.09972
47B7	0.70228	47B7	0.33399	0.09866
**Mean**	0.81802	**Mean**	0.41891	0.05211
**Median**	0.83982	**Median**	0.36331	0.05051

Table S8. AUCs for each individual in fine-tuned models, compared to the original

**Before tuning**		**After tuning**	
**Patient**	**Ensemble model**	**Patient**	**Ensemble model**
02CE	0.91557	02CE	0.94974
0D51	0.81612	0D51	0.85828
0FA7	0.93425	0FA7	0.96981
11FD	0.90045	11FD	0.97437
16A9	0.87588	16A9	0.93033
1B55	0.72626	1B55	0.75031
2257	0.51525	2257	0.92549
32B1	0.9567	32B1	0.96611
2BAF	0.88358	2BAF	0.75974
305B	0.70921	305B	0.89419
4561	0.68379	4561	0.68484
47B7	0.78719	47B7	0.80158
**Mean**	0.80869	**Mean**	0.87207
**Median**	0.84600	**Median**	0.90984

Table S9. AUPRCs for each individual in fine-tuned models, compared to the original

Before tuning		After tuning		
**Patient**	**ensemble (mean)**	**Patient**	**ensemble (mean)**	**baseline**
02CE	0.58943	02CE	0.72932	0.03155
0D51	0.24475	0D51	0.39015	0.04892
0FA7	0.5848	0FA7	0.70049	0.04772
11FD	0.35281	11FD	0.57688	0.02037
16A9	0.40664	16A9	0.68328	0.03265
1B55	0.22112	1B55	0.19149	0.06719
2257	0.07368	2257	0.68609	0.04005
32B1	0.63705	32B1	0.79834	0.02046
2BAF	0.36833	2BAF	0.37596	0.03865
305B	0.37801	305B	0.61791	0.09417
4561	0.43477	4561	0.49435	0.17555
47B7	0.2792	47B7	0.51653	0.08756
**Mean**	0.38088	**Mean**	0.56340	0.05874
**Median**	0.37317	**Median**	0.59740	0.04389

Table S10. AUCs for aggregation on the entire meals

**Model**	**Mean**	**Median**
Data: Accelerometer	0.9103	0.9100
Data: Gyroscope	0.9478	0.9535
Data: Accelerometer + Gyroscope	0.9215	0.9335
Normalization: Z-score	0.9495	0.9459
Normalization: Centering	0.9326	0.9280
Augmentation: Quaternion rotation	0.9370	0.9361
Augmentation: Magnitude scaling	0.9506	0.9475

Augmentation: Quaternion rotation + Magnitude scaling	0.9350	0.9414

Table S11. Accuracies for the aggregation on the 5- and 10-minute meals after meal start

Evaluation Thresholds	Meal Length	Required Counts	Accuracy
0.1	5	1	0.9365
0.2	5	1	0.8413
0.3	5	1	0.7937
0.4	5	1	0.7460
0.5	5	1	0.6984
0.6	5	1	0.6825
0.7	5	1	0.5556
0.8	5	1	0.4762
0.9	5	1	0.3492
0.1	5	3	0.8730
0.2	5	3	0.7460
0.3	5	3	0.7143
0.4	5	3	0.6825
0.5	5	3	0.6190
0.6	5	3	0.5079
0.7	5	3	0.4444
0.8	5	3	0.2857
0.9	5	3	0.2063
0.1	10	1	0.9841
0.2	10	1	0.9206
0.3	10	1	0.9206
0.4	10	1	0.8730
0.5	10	1	0.8254
0.6	10	1	0.7937
0.7	10	1	0.7143
0.8	10	1	0.5873
0.9	10	1	0.5079
0.1	10	3	0.9683

0.2	10	3	0.9048
0.3	10	3	0.8889
0.4	10	3	0.7937
0.5	10	3	0.7302
0.6	10	3	0.6508
0.7	10	3	0.5556
0.8	10	3	0.4444
0.9	10	3	0.3175

Table S12. False-positive alarms per hour for the aggregation on the all negative regions

Evaluation Thresholds	Required Counts	False Positive Alarm Per Hour
0.1	1	1.7303
0.2	1	0.8622
0.3	1	0.3507
0.4	1	0.1783
0.5	1	0.1140
0.6	1	0.0555
0.7	1	0.0292
0.8	1	0.0146
0.9	1	0.0088
0.1	3	0.8739
0.2	3	0.3887
0.3	3	0.1724
0.4	3	0.0965
0.5	3	0.0438
0.6	3	0.0205
0.7	3	0.0146
0.8	3	0.0117
0.9	3	0.0058

Table S13. Network structures

Layer	Type	# of filters	Kernel size	Stride	Padding	Activation	Output size*
input_1	Input	/	/	/	/	/	(None, 15000, 3)
Conv1d_1	Convolution	8	11	1	Valid	ReLU	(None, 14990, 8)
BN_1	Batch Normalization	/	/	/	/	/	(None, 14990, 8)
MaxPool_1	Max Pooling	/	2	2	Valid	/	(None, 7495, 8)
Conv1d_2	Convolution	16	10	1	Valid	ReLU	(None, 7486, 16)
BN_2	Batch Normalization	/	/	/	/	/	(None, 7486, 16)
MaxPool_2	Max Pooling	/	2	2	Valid	/	(None, 3743, 16)
Conv1d_3	Convolution	16	10	1	Valid	ReLU	(None, 3734, 16)
BN_3	Batch Normalization	/	/	/	/	/	(None, 3734, 16)
MaxPool_3	Max Pooling	/	2	2	Valid	/	(None, 1867, 16)
Conv1d_4	Convolution	32	8	1	Valid	ReLU	(None, 1860, 32)
BN_4	Batch Normalization	/	/	/	/	/	(None, 1860, 32)
MaxPool_4	Max Pooling	/	2	2	Valid	/	(None, 930, 32)
Conv1d_5	Convolution	32	9	1	Valid	ReLU	(None, 922, 32)
BN_5	Batch Normalization	/	/	/	/	/	(None, 922, 32)
MaxPool_5	Max Pooling	/	2	2	Valid	/	(None, 461, 32)
Conv1d_6	Convolution	64	6	1	Valid	ReLU	(None, 456, 64)
BN_6	Batch Normalization	/	/	/	/	/	(None, 456, 64)
MaxPool_6	Max Pooling	/	2	2	Valid	/	(None, 228, 64)

Conv1d_7	Convolution	64	7	1	Valid	ReLU	(None, 222, 64)
BN_7	Batch Normalization	/	/	/	/	/	(None, 222, 64)
MaxPool_7	Max Pooling	/	2	2	Valid	/	(None, 111, 64)
Conv1d_8	Convolution	128	4	1	Valid	ReLU	(None, 108, 128)
BN_8	Batch Normalization	/	/	/	/	/	(None, 108, 128)
MaxPool_8	Max Pooling	/	2	2	Valid	/	(None, 54, 128)
Conv1d_9	Convolution	128	5	1	Valid	ReLU	(None, 50, 128)
BN_9	Batch Normalization	/	/	/	/	/	(None, 50, 128)
MaxPool_9	Max Pooling	/	2	2	Valid	/	(None, 25, 128)
Conv1d_10	Convolution	256	2	1	Valid	ReLU	(None, 24, 256)
BN_10	Batch Normalization	/	/	/	/	/	(None, 24, 256)
MaxPool_10	Max Pooling	/	2	2	Valid	/	(None, 12, 256)
flatten_1	Flatten	/	/	/	/	/	(None, 3072)
dense_1	Dense	/	/	/	/	Sigmoid	(None, 1)

*The positions of “None” are for the batch size

Table S14. DeepConvLSTM structures

Layer	Type	# of filters	Kernel size	Stride	Activation	Output size*
input_1	Input	/	/	/	/	(None, 20, 15000, 3)
Conv1d_1	Convolution	32	(1, 5)	1	ReLU	(None, 20, 14996, 32)
BN_1	Batch Normalization	/	/	/	/	(None, 20, 14996, 32)
MaxPool_1	Max Pooling	/	(1, 2)	(1, 2)	/	(None, 20, 7498, 32)
Conv1d_2	Convolution	32	(1, 5)	1	ReLU	(None, 20, 7494, 32)
BN_2	Batch Normalization	/	/	/	/	(None, 20, 7494, 32)
MaxPool_2	Max Pooling	/	(1, 2)	(1, 2)	/	(None, 20, 3747, 32)
Conv1d_3	Convolution	32	(1, 5)	1	ReLU	(None, 20, 3743, 32)
BN_3	Batch Normalization	/	/	/	/	(None, 20, 3743, 32)
MaxPool_3	Max Pooling	/	(1, 2)	(1, 2)	/	(None, 20, 1871, 32)
Conv1d_4	Convolution	32	(1, 5)	1	ReLU	(None, 20, 1867, 32)
BN_4	Batch Normalization	/	/	/	/	(None, 20, 1867, 32)
MaxPool_4	Max Pooling	/	(1, 2)	(1, 2)	/	(None, 20, 933, 32)
reshape_1	Reshape	/	/	/	/	(None, 20, 29856)
lstm_1	LSTM**	/	/	/	Tanh	(None, 20, 64)
lstm_2	LSTM**	/	/	/	Tanh	(None, 20, 64)
dense_1	Time distributed dense	/	/	/	Sigmoid	(None, 20, 1)
reshape_2	Reshape	/	/	/	/	(None, 20)

*The positions of “None” are for the batch size

**Use dropout = 0.5

Table S15. Examples of other types of instruments in studying digital biomarkers for eating behavior.

Study	Definitions of eating	Device position	Number of participants	Total hours	Weighted accuracy
Nishimura et al [28]	Eating habits monitoring using wireless, wearable in-ear microphone	Ear	N/A[a]	• 100 chews for each food sample: potato chips, rice, banana, salad, wafers, apples, peanuts, jelly, rice crackers.<br>• Data were approximately 1 minute in length.	Average error rate 1.93%
Ravi et al [29]	Real-time food intake classification and energy expenditure estimation on a mobile device	Image-based classification	5	• The data set UEC-FOODI00 contained 100 food categories with >100 images per category.<br>• The total number of food images in the data set was 12,905.	Up to 0.78 accuracy
Liu et al [30]	An intelligent food intake monitoring system using wearable sensors	Ear microphone and camera	6	• Lunch up to 30 minutes	Eating recognition rate 82.51%
Mirtchouk et al [5]	Recognizing eating from body-worn sensors: combining free-living and laboratory data	Google Glass for head motion, smartwatches on each wrist for wrist motion, and an earbud to capture chewing sounds	6 in laboratory study and 6 new to test; generalizability	• Laboratory: 72 hours<br>• Free-living: 180 hours	Leave one free-living session out: precision 31%; recall 87%; accuracy 85%

Gao et al [31]	iHearFood: eating detection using commodity Bluetooth headsets	Bluetooth headsets	28	• 20 hours	Leave one sample out 94.72%; leave one person out 76.82%
Blechert et al [32]	Unobtrusive electromyography-based eating detection in daily life: a new tool to address underreporting?	Electromyography sensors	15	• 360 hours	Sensitivity mean 87.3 (SD 21.7) and specificity mean 86.9 (SD 16.8)

[a]N/A: not available.
